# The Emerging Role of Presepsin (P-SEP) in the Diagnosis of Sepsis in the Critically Ill Infant: A Literature Review

**DOI:** 10.3390/ijms222212154

**Published:** 2021-11-10

**Authors:** Chiara Maddaloni, Domenico Umberto De Rose, Alessandra Santisi, Ludovica Martini, Stefano Caoci, Iliana Bersani, Maria Paola Ronchetti, Cinzia Auriti

**Affiliations:** 1 Neonatal Intensive Care Unit (NICU), Medical and Surgical Department of the Fetus—Newborn-Infant, “Bambino Gesù” Children’s Hospital IRCCS, 00165 Rome, Italy; chiara.maddaloni@opbg.net (C.M.); domenico.derose@opbg.net (D.U.D.R.); alessandra.santisi@opbg.net (A.S.); ludovica.martini@opbg.net (L.M.); stefano.caoci@opbg.net (S.C.); iliana.bersani@opbg.net (I.B.); mariapaola.ronchetti@opbg.net (M.P.R.); 2 Neonatal Intensive Care (NICU) and Neonatal Pathology, San Vincenzo Hospital, 98039 Taormina, Italy

**Keywords:** sepsis, infants, biomarkers, neonate, septic shock, point-of-care

## Abstract

Sepsis causes high rates of morbidity and mortality in NICUs. The estimated incidence varies between 5 and 170 per 1000 births, depending on the social context. In very low birth-weight neonates, the level of mortality increases with the duration of hospitalization, reaching 36% among infants aged 8–14 days and 52% among infants aged 15–28 days. Early diagnosis is the only tool to improve the poor prognosis of neonatal sepsis. Blood culture, the gold standard for diagnosis, is time-consuming and poorly sensitive. C-reactive protein and procalcitonin, currently used as sepsis biomarkers, are influenced by several maternal and fetal pro-inflammatory conditions in the perinatal age. Presepsin is the N-terminal fragment of soluble CD14 subtype (sCD14-ST): it is released in the bloodstream by monocytes and macrophages, in response to bacterial invasion. Presepsin seems to be a new, promising biomarker for the early diagnosis of sepsis in neonates as it is not modified by perinatal confounding inflammatory factors. The aim of the present review is to collect current knowledge about the role of presepsin in critically ill neonates.

## 1. Introduction

Sepsis causes high rates of morbidity and mortality in Neonatal Intensive Care Units (NICUs). In very-low birth-weight neonates (VLBW), early-onset sepsis (EOS)-related mortality has been reported to be around 40%, while late-onset sepsis (LOS)-related mortality ranges from 2% to 50%, depending on the aggressiveness of the pathogen, the length of the hospitalization and the age of the neonate [1,2]. Moreover, in extremely fragile neonates, sepsis significantly impairs long-term outcomes if it is not diagnosed and treated promptly [3].

The early diagnosis of sepsis in neonates can be a challenge because of its unspecific clinical presentation, the low sensitivity of blood culture, and the poor performance of the currently used markers of infection during the first days of life [4]. Serum levels of C-reactive protein (CRP) and procalcitonin (PCT) show a physiological increase during the first 48–72 h of life and are influenced by several maternal and fetal pro-inflammatory conditions, other than infections [5].

Although early empiric broad-spectrum antibiotics are recommended in case of a suspected infection, antibiotic de-escalation should also be a goal and may be a safe practice in case of negative cultures [6], considering the increased risk of necrotizing enterocolitis and/or death when antibiotics are unnecessary [7,8]. Indeed, emerging multi-drug-resistant (MDR) pathogens emphasize the urgent need to reduce antibiotics use and control their further spread [9]. For these reasons, the development of a rapid and accurate diagnostic test with a strong negative predictive value of sepsis is crucial to reduce abuse of antibiotics in neonates [10]. The ideal marker for infection should be valuable for establishing the diagnosis, as well as for predicting the outcome and for evaluation of the response to treatment; concomitantly, it should be easy to quantify and available for routine clinical use [11].

Presepsin (P-SEP) is an immunologic biomarker that offers a good accuracy in detecting different infections in adults [12]. However, there are some uncertainties as to its accuracy in the diagnosis of neonatal sepsis, mainly concerning the immaturity of the kidney function of neonates, especially those born preterm, which could influence the choice of the best cut-off, the type of sepsis, and early-onset or late-onset, which feature different characteristics in neonates. Although current results should be carefully read and few studies with adequate sample sizes are currently available, P-SEP seems to be a promising marker for diagnosis of sepsis in neonates [13]. The aim of this review is to summarize current evidence about the diagnostic accuracy of presepsin in the neonatal age.

## 2. Materials and Methods

This review was produced by searching for articles in the PubMed database and matching the terms “presepsin” and “neonate” or “newborn”. All retrieved articles written in English and published before 1 October 2021 were analyzed, without imposing restrictions on date or year, locations, study design, study aim, or inclusion/exclusion criteria. We screened the reference lists of the identified studies and additional references for this review were identified by each author based on their knowledge on the field. We also provide a brief overview about the biological characteristics of presepsin and its diagnostic accuracy in adults and children.

## 3. Results

A total of 53 records were identified through the literature search (via PubMed), published from 2010 onwards. Among them, 35 were excluded based on the titles, the abstracts, and the type of study. A further seven articles were identified through a literature search via Google.

Twenty-one full-text articles were eventually included in a qualitative synthesis of current knowledge of P-SEP levels in neonates: three studies on the reference ranges in neonatal age [4,14,15], four studies on the diagnosis of early-onset sepsis (EOS) [10,16,17,18], four studies on the diagnosis of late-onset sepsis (LOS) [1,19,20,21] and ten studies on the diagnosis of neonatal sepsis in general (EOS and LOS considered together) [22,23,24,25,26,27,28,29,30,31].

We included separately a qualitative synthesis, including four were meta-analyses that summarized in a quantitative way the results of this particularly interesting topic [32,33,34,35].

## 4. Biological Characteristics of Presepsin

CD14 is a glycoprotein, a member of Toll-like receptor (TLR) family, constitutively expressed on the cell surface of monocytes/macrophages [12,36]. The principal ligand of CD14 is bacterial lipopolysaccharide (LPS), which when associated with Lipoprotein Binding Protein (LBP) forms a complex easily recognized by the TLR. CD14 takes two forms: membrane-bound CD14 (mCD14) and soluble CD14 (sCD14). The latter is seen in plasma and is produced by mCD14 fall-off or cell secretion [10]. Presepsin is the N-terminal fragment of 13 kDa of soluble CD14 subtype (sCD14-ST).

The binding between CD14 and LPS-LBP complex activates the TLR4-specific proinflammatory signaling cascade, playing a role in the identification of several Gram-positive and Gram-negative bacterial ligands and leading to the release of cytokines (tumor necrosis factor-α, IFN-γ, IL-1β, IL-8 and IL-6) [11].

**Figure 1:**
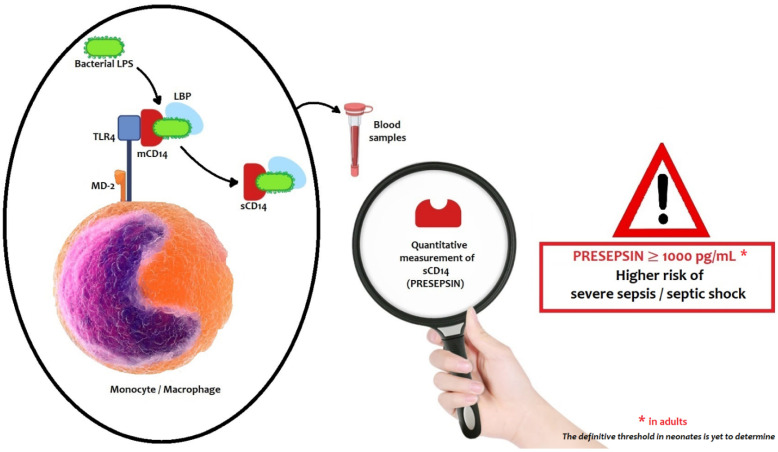
The mechanism of presepsin production.

After TLR4 activation, the complex LPS-LBP-CD14 is internalized into a phagolysosome in monocyte-macrophage cells. The CD14 glycoprotein is cleaved by cathepsin D and other proteases in plasma or in the phagolysosome. After proteolysis, the N-terminal fragment of 64 amino acids, named presepsin (P-SEP), is released in the bloodstream by exocytosis (Figure 1) [12].

The biological function of presepsin, beyond infectious episodes, is currently under investigation. It has been reported that P-SEP promotes phagocytosis, and its plasmatic levels depend on the intensity of the innate immune response. The recognition of P-SEP is possible even in healthy uninfected individuals; nevertheless, its levels markedly increase during the early phases of bacterial infections, and correlate with treatment efficacy and prognosis [37].

Little is known about P-SEP and fungal bloodstream infections. A single-center study investigated the relationship between P-SEP plasmatic levels and fungemia, performing an additional in vitro study to compare the increase in P-SEP levels between fungi and bacteria [38]. P-SEP was increased in all patients with fungal bloodstream infections, with a significant correlation between plasmatic concentrations and the SOFA (Sequential Organ Failure Assessment) score (r = 0.89, *p* < 0.001). An in vitro assay with the co-incubation of Candida albicans and human whole blood cells indicated that the viable cells of C. albicans caused an increase in presepsin, as seen with Escherichia coli [38]. Further multi-centric studies are needed to confirm these preliminary results about P-SEP as marker of fungemia.

## 5. Presepsin Plasmatic Levels and Renal Function

Renal function plays an important role in the evaluation of plasma biomarkers, since their concentration largely depends on renal excretion. The plasma concentration of P-SEP seems to increase with renal impairment, as it is a 13 kDa protein, similar to Cistatin C, a protein that is filtered by the glomerulus and provides an indication as to kidney function. It is presumed from its molecular weight that, when filtered by the glomerulus, P-SEP is reabsorbed and catabolized within proximal tubular cells. Therefore, any disease affecting kidney filtration could affect the plasmatic concentration of P-SEP, causing its plasmatic levels to rise [39].

P-SEP levels are inversely correlated with Glomerular Filtration Rate (GFR). In particular, in patients receiving hemodialysis, P-SEP values were markedly high, at comparable levels to those seen in severe sepsis or septic shock, and returned to normal levels after kidney transplantation [40,41,42].

In very low birth-weight infants, renal function is often compromised by prematurity, regardless of the presence of infection. High levels of P-SEP due to impaired kidney function could lead to the misdiagnosis of sepsis. To date, the relationships between renal function and plasma levels of P-SEP have been studied in adults and children [43], but not yet in neonates. In adults, high levels of P-SEP in uninfected patients with severe acute kidney failure have been reported in previous studies and higher cut-offs have been established to improve the test accuracy in discriminating sepsis [44,45].

In a single-center retrospective study on a group of 366 patients with acute kidney failure and another of 440 patients with normal kidney function, the optimal cut-off values in normal patients were 240 pg/mL (sensitivity: 80.9%, specificity: 83.2%) for P-SEP, and 0.10 ng/mL (sensitivity: 85.1%, specificity: 79.1%) for PCT. In patients with severe kidney failure, the optimal cut-off values were 500 pg/mL (sensitivity: 89.7%, specificity: 59.7%) for the P-SEP, and 4.07 ng/mL (sensitivity: 87.2%, specificity: 93.5%) for PCT. Thus, the optimal cut-off values of P-SEP and PCT were higher in the group of patients with severe kidney failure compared to those of normal patients. However, the study was conducted only on patients aged ≥18 years with no reference to neonates [46].

Concerning neonates, the available research is still poor. In a case report of a preterm with nephrogenic diabetes insipidus, persistently elevated concentrations of P-SEP in the absence of sepsis were reported (range from 668 to 1647 pg/mL) [47].

It will likely be necessary to identify, with further studies, specific cut-off levels higher than those currently identified in neonates and infants, involving those who have an underlying disease and impaired renal function.

In the study carried out by Pietrasanta et al. on 159 preterm infants with suspected sepsis (58 of them with infection, 77 with sepsis and 24 with septic shock) P-SEP levels were elevated in neonates with septic shock (median 1557.5 pg/mL) and sepsis (median 1361 pg/mL) compared with those detected in neonates with other infections [31].

It is possible that in infants with septic shock, some of these levels may be attributable to the effect of shock on the kidney function and therefore the cut-off in these cases should be better studied.

## 6. Methods to Measure Presepsin Plasma Levels

Different methods have been developed to measure presepsin (P-SEP). Firstly, a traditional two-step sandwich enzyme-linked immunosorbent assay (ELISA) was used for the detection of presepsin by the recombinant CD14 (S286C) as standard with results in 4 h. This assay lacked speed and accuracy, which are essential for routine assessments in intensive care units (ICUs). A few years later, a one-step ELISA assay was developed using recombinant presepsin and two new anti-P-SEP antibodies. As a result, the total analysis time was reduced from 4 h to 1.5 h, with the same performance characteristics as the two-step assay [48].

A step forward in the plasmatic measure of P-SEP is represented by a novel, highly sensitive and fully automated method, based on the chemiluminescence (CLEIA) method, providing results in 17 min in six samples simultaneously by a Point-of-Care Testing (POCT) instrument [49,50]. The test is based on a non-competitive CLEIA combined with MAGTRATION^®^ technology (Precision System Science/PSS USA Inc., Livermore, CA, USA). The term ‘‘Magtration’’ is an abbreviation of “Magnetic Filtration” and is based on bound/free separation in pipette tips using magnetic particles [51].

Magnetic particles were coated by an anti-P-SEP polyclonal antibody and a monoclonal antibody. During incubation with plasma, they formed immunocomplexes with P-SEP present in the sample. A chemiluminescent substrate was added. After a short incubation, the luminescence intensity generated by the enzyme reaction was measured. The luminescence intensity was directly correlated to the P-SEP concentration in the sample, which was calculated by means of the standard curve [49].

The CLEIA presepsin assay correlated well with a previously reported two-step presepsin ELISA. According to the results from Okamura et al. [40], the limit of blank, the limit of detection, and the limit of quantification were 2.33, 13.4, and 47.6 pg/mL, respectively. The assay linearity was achieved up to 20,000 pg/mL. The intra-assay imprecision was 3.4–4.8% for plasma and 2.7–7.1% for whole blood. The within-run imprecision and total imprecision for plasma were 3.6–4.4% and 5.2–6.5%, respectively. No interference of presepsin was detected with other analytes, such as bilirubin, hemoglobin, lipids, triglycerides, or rheumatoid factors [40].

This approach performed effectively enough to be applicable for use in the Emergency Department (ED), ICUs, and the surgical wards; it is currently preferred in both clinical activity and research.

## 7. Accuracy of Presepsin in Detecting Sepsis in Adults and Children

According to the results of several multicenter prospective studies, P-SEP levels are significantly higher in patients with systemic bacterial infections than in those with Systemic inflammatory response syndrome (SIRS) or other diseases [52,53,54]. The cut-off value of 600 ng/L has been reported for the discrimination of bacterial sepsis with a sensitivity and specificity of 87.8% and 81.4%, respectively [52]. Higher levels of P-SEP have been reported in patients with Gram-negative bacterial infections [52,53].

Presepsin values are usually interpreted as follows [55]:

− Presepsin < 200 pg/mL—sepsis excluded;
− Presepsin < 300 pg/mL—systemic infection improbable;
− Presepsin < 500 pg/mL—sepsis probable;
− Presepsin < 1000 pg/mL—significant risk of severe sepsis;
− Presepsin ≥ 1000 pg/mL—high risk of severe sepsis/septic shock equivalent to a SOFA score ≥ 8.

Changes in presepsin values may be an appropriate indicator for monitoring antibiotic therapy: in fact, it tended to reduce on day 7, in patients with positive blood cultures and appropriate antibiotic therapy, and it rose in those with inappropriate antibiotic therapy [53].

In an observational prospective study, a presepsin assay with a cutoff at 588 ng/L showed high level of sensitivity (81%) and specificity (80%) in the diagnosis of community-acquired pneumonia (CAP). In these patients, presepsin levels higher than 556 ng/L showed significantly lower survival rates (50.0% vs. 76.6%) [56].

Recent studies suggested that the use of new generation biomarkers, such as presepsin, alongside integral severity-of-disease scores allow the prediction of the risk of infectious complications and mortality in cardio-surgical patients. Similarly, presepsin seems to be as valuable a biomarker as PCT or CRP in the evaluation of infectious complications in patients after heart transplantation [41].

Few studies are available on the efficacy of P-SEP as a diagnostic biomarker for sepsis in children. A recent paper from Sakyi et al. explored the concept of a Bioscore (a combination of three biomarkers: CRP, PCT and presepsin) that seemed to be more efficient in the identification of pediatric sepsis. In fact, with at least two of the three markers above their respective threshold (Bioscore 2 or 3), a greater proportion (>75%) of cases were shown to have sepsis [57].

According to a meta-analysis by Yoon et al., P-SEP offers higher sensitivity and diagnostic accuracy, but lower specificity, when compared to PCT or CRP in detecting sepsis in children. However, these results must be carefully interpreted as the number of studies included was small and the studies were statistically heterogeneous [58].

## 8. Ranges of Reference Values in Neonates

The availability of reference values of P-SEP in term and preterm neonates is crucial for obtaining an adequate diagnostic accuracy to rule out sepsis. Since 2012, different groups have investigated this topic, but due to small sample sizes and the type of study (case-control) they have found extremely variable and unreliable results.

Pugni et al., in Italy, conducted the first study properly designed to identify reference ranges of P-SEP in neonates [14]. Of the 684 neonates enrolled in their study, 484 (70.8%) were born at term and 200 (29.2%) were preterm (24–36 weeks’ gestation). For each neonate, maternal and perinatal variables connected with inflammation were collected (intra-partum fever, premature rupture of the membranes, delivery mode, perinatal asphyxia, meconium aspiration, respiratory distress syndrome, surfactant administration and prenatal steroids administration). The mean age at blood sampling was 3.6 ± 0.6 days in term infants and 3.9 ± 0.8 days in preterm infants. The distribution of percentiles of presepsin levels (pg/mL) in term and preterm neonates enrolled in their study are shown in Table 1. The P-SEP values were found to be slightly higher in preterm than in at-term neonates, and higher in neonates than in the healthy adult population. No significant differences were demonstrated in preterm infants at different gestational ages. Among the variables that could modify the P-SEP values in the group of term infants, only the low Apgar score at 1 min was significantly associated with P-SEP plasma levels (*p* = 0.032). No differences in presepsin values were observed between weight loss at blood sampling 10 and >10%.

Concerning only preterm infants born before 32 weeks of GA, Poggi et al. conducted in 2020 a multicenter study enrolling them separately from other preterm infants, determining the P-SEP levels during the first 48 h of life and excluding cases of EOS [4].

The distribution of values (in pg/mL) in enrolled preterm neonates is also reported in Table 1. Among the analyzed variables, Poggi et al. found that only GA affected presepsin values in the first 24 h of life. Presepsin in the first six hours of life seemed to decrease on average by 29 ng/L for each week of GA increase. After 48 h of life, P-SEP was not more correlated with GA, which was in agreement with the data obtained by Pugni in 2015. Furthermore, other variables (such as birthweight, white blood cells, delivery mode, steroids within three days prior delivery, premature rupture of the membranes and intrapartum prophylaxis) were confirmed to not influence P-SEP levels [4]. Other authors found no correlation between presepsin levels and gestational age, further suggesting that P-SEP is not affected by postnatal age [10].

In another, Japanese study, plasma P-SEP levels were examined in 30 healthy term neonates at birth, on the first day and on the fifth day of life. The levels on the fifth day of life were lower than before (*p* < 0.001). The authors hypothesized that para-physiological and transient renal dysfunction may be involved in elevating serum P-SEP in the first day of life [15].

According to the available research, neonatal reference ranges are substantially higher than those seen in healthy adults. Among the possible explanations, we count the activation of the innate immune system occurring after birth, as a result of the transition from the normally sterile intra-uterine environment to a world rich in foreign antigens. Furthermore, following birth, the neonatal skin and gut are rapidly colonized with microbial flora, representing a continuous stimulus to the innate immune system [59].

In agreement with other authors, Levy et al. demonstrated that preterm and full-term neonates express significantly greater CD14 expression on peripheral blood monocytes, both at baseline and after LPS stimulation, compared with adults [60].

## 9. Presepsin Compared to Other Immunologic Biomarkers

C-reactive protein (CRP) and procalcitonin (PCT) are currently used as infection biomarkers; nevertheless, they change considerably during the early neonatal period due to several non-infectious conditions [15].

CRP is an acute-phase protein synthesized in the liver in response to inflammatory and infectious stimuli. The first CRP increase after induction occurs at about 12 h and reaches its peak after around 20–72 h. Several confounding variables are associated with CRP increase in the healthy neonate: delivery mode (vaginal delivery versus cesarean section), a longer duration of active labor and possibly intrapartum fetal distress [41]. Furthermore, CRP rises by 6.0% per week of GA at delivery, 2.4% for each growth of 100 g of birth weight, 0.4% for each hour of preterm premature rupture of the membranes (pPROM), 40% in the case of antenatal steroids and 28% in the case of intrapartum antibiotic prophylaxis [5]. A cut-off value >10 mg/L for CRP was shown to be the most appropriate in neonates.

The early rise in CRP levels in neonates after birth usually leads to the suspicion of an EOS and to the commencement of empiric antibiotics, although CRP may increase up to 20 mg/L during the first days of life even in uninfected newborns [61]. Indeed, empiric antibiotics should not be administered if the decision is based solely on the CRP levels obtained in first days of life, which could be similar both in infants with positive blood cultures and in infants with negative blood cultures [6]. CRP measurements are meaningful only when framed in the context of specific risk factors and clinical signs and symptoms concordant with the suspicion of EOS [62].

In conclusion, CRP specificity is low, and it is preferably used in combination with another serum biomarker.

PCT is a prohormone of calcitonin. In healthy individuals, it is produced in thyroid C cells, from a calcitonin gene-related peptide I (CALC-1) located on chromosome 11. The mRNA product is known as pre-procalcitonin, and it is further modified to 116 amino acid procalcitonin, and all the PCT formed in thyroid C cells is converted to calcitonin. In healthy subjects, plasma levels of PCT are very low (0.05 ng/mL) [63]. In the course of bacterial infection, serum levels start to increase after 2–4 h of bacterial endotoxin release, peaking after 6–8 h and returning to normal levels after 24 h [40]. PCT values increase earlier than CRP values in severe infections and also fall quicker after appropriate antibiotic treatment [5]. In contrast to CRP, local bacterial infections, severe viral infections and inflammatory reactions of non-infectious origin are only associated with a slight increase in PCT [41].

In healthy newborns, PCT levels show a physiologic increase 24–48 h after birth and decreases to normal levels after 3 days. Moreover, the mean PCT decreases by 11.4% per week of GA at delivery, and by 2.2% per 100 g of weight independently of gender. PCT levels at birth could be modified in the case of pPROM, clinical chorioamnionitis and gestational diabetes [5]. For PCT, 0.5–2 ng/mL may be the appropriate cut-off interval in newborns [33].

An interesting metanalysis compared the diagnostic accuracy of PCT, CRP, procalcitonin combined with C-reactive protein and presepsin in the diagnosis of neonatal sepsis, including 28 studies in which 2661 patients were enrolled. The authors found that, although PCT is more sensitive than CRP, the use of PCT or CRP alone cannot rule out a diagnosis of neonatal sepsis. In fact, only the combination of PCT and CRP or presepsin alone improves the accuracy of diagnosis of neonatal sepsis. The AUC for presepsin (0.99) was greater than PCT + CRP (0.96), CRP (0.85) and PCT (0.91). From the subgroup analysis, the same authors established suitable cut-off values for PCT (0.5–2 ng/mL) and CRP (>10 mg/L) [33].

Presepsin rises after 2 h, with a peak at about 3 h after onset of the infection, and a decline after 4–8 h [64]. As previously mentioned, serum presepsin levels are not affected by common perinatal factors [1,10]. Recent studies indicate that P-SEP can be used to monitor clinical response to therapeutic interventions prior to obtaining culture results, confirming it as a more reliable biomarker than CRP and PCT in neonatal sepsis [1,26,27]. The sensitivity and specificity of P-SEP obtained at a cutoff level of 722 μg/L seems to be higher than its sensitivity and specificity at a cutoff level of 539 μg/L [33].

## 10. Presepsin for the Detection of Early-Onset and Late-Onset Sepsis

The early diagnosis of bacterial sepsis in neonates is hampered by non-specific symptoms and the lack of rapid responding laboratory measures. The gold standard diagnostic test is blood culture (BC). According to Kuzniewicz’s findings, pathogens are usually isolated by 36 h after BC collection [65], but microbiological isolation is, unfortunately, time-consuming. An early, sensitive and specific laboratory test would be helpful to guide clinicians to decide whether or not to start antibiotics, avoiding unnecessary treatment. Presepsin could be the appropriate marker.

Early-onset sepsis (EOS) reflects transplacental and ascending infections from the maternal genital tract within 72 h after birth. Late-onset sepsis (LOS) (≥72 h after birth) is associated with the nosocomial environment, affecting preterm and VLBW neonates in particular [1]. It has been reported that an ideal biomarker for neonatal sepsis should offer a sensitivity of 100%, specificity > 85%, a positive predictive value > 85% and a negative predictive value of 100% [64].

In Table 2, we report all the available studies on the reference ranges of P-SEP levels at neonatal age and on the P-SEP levels in the case of EOS, LOS and both (EOS and LOS together).

We also identified four meta-analyses that summarized in a quantitative way the results of the accuracy of P-SEP and the pooled sensitivity and specificity obtained including P-SEP evaluation (Table 3).

Recently, van Maldeghem et al. evaluated the differences in the diagnostic accuracy of presepsin between EOS and LOS: 12 articles were included in the systematic review and 10 in the meta-analysis [34]. The optimal cut-off values ranged from 305 to 672 ng/L for the EOS cases versus the healthy controls, with a pooled sensitivity of 81% (95%CI: 0.76–0.85), a pooled specificity of 86% (0.81–0.89) and an area under the curve (AUC) of 0.9412 (SE 0.1178).

Differences in presepsin levels were seen between EOS and LOS: in LOS, the optimal cut-off values were higher, ranging from 801 to 885 ng/L, with a pooled sensitivity of 81% (0.74–0.86) and a pooled specificity of 100% (0.98–1.00). The AUC was not estimable in the LOS cases because of the low number of studies. The hypothesis is that the sepsis evaluation in EOS is performed in the early ‘pre-clinical’ stage of the infection, whereas presepsin in LOS is analyzed at the clinical stage, when the symptoms of infection are already present.

Considering all the sepsis cases together (EOS, LOS and combined), the cut-off values ranged from 305 to 885 ng/L. The pooled sensitivity was 92% (0.91–0.93) and the pooled specificity was 86% (0.84–0.87), with an AUC of 0.9639 (0.0181) [34].

According to the results obtained by Pietrasanta et al., P-SEP levels correlate with the severity of disease: they were higher in neonates with septic shock (median 1557.5 pg/mL) and sepsis (median 1361 pg/mL) compared to those with infection (median 977.5 pg/mL) at the point at which sepsis was first suspected (*p* < 0.01) [31].

Furthermore, as shown in three studies, serum presepsin levels decrease progressively in the course of the antibiotic treatment of both EOS and LOS, suggesting its possible role in monitoring therapeutic response [1,19,27,28,30].

So far, the cut-off values, together with the specificity and sensitivity, have varied greatly among studies and the type of infection (EOS vs. LOS) and sampling time [32].

## 11. Prognostic Significance of Presepsin

Elevated plasma P-SEP values appear to be predictive of sepsis severity and death. In a 2018 meta-analysis that included a total of 1617 adult patients, Yang et al. reported that P-SEP levels in the first 24 h were significantly higher in non-survivors [66].

The relationship between plasma P-SEP levels and mortality was explored in 31 neonates and the authors found a significant positive correlation between P-SEP levels measured on the first day of life and the highest sepsis severity score [67]. In an interesting study by Astrawinata et al. [21], early P-SEP values were significantly higher in the non-survivor group than in the survivor group (*p* < 0.05), when compared to CRP and PCT.

This relationship is less clearly established among pediatric patients. El Gendy et al. studied 80 children with an average age of 14 months who had been admitted to a pediatric intensive care unit due to infection. The infants with sepsis had higher presepsin levels than the healthy controls (*p* = 0.0001) but no difference was found between the survivors and non-survivors (*p* = 0.84). However, higher P-SEP levels were associated with higher rates of mechanical ventilation (*p* = 0.048) as well as longer hospital stays (*p* = 0.012), suggesting that P-SEP levels are an appropriate indicator of disease severity [68].

P-SEP seems to be a useful biomarker in the early diagnosis and prognosis of neonatal sepsis; however, data in this particularly interesting field remain too limited to reach definitive conclusions.

## 12. Conclusions

Neonatal sepsis is a fearful condition with a great impact in terms of morbidity and mortality. Obtaining a ‘magical’ biological marker with maximum sensitivity and maximum negative predictive values would help to prevent the serious consequences of sepsis in many newborns. Although CRP and PCT are the most widely used biomarkers of neonatal sepsis, their accuracy is still controversial. There is emerging evidence about the role of presepsin as a precocious marker of sepsis in neonates. Based on current clinical study results, most factors affecting CRP and PCT levels seem not to affect presepsin levels.

P-SEP has shown good accuracy in diagnosing both neonatal EOS and LOS, even if the data are still limited. There is a wide heterogeneity among the relevant studies in terms of the onset of sepsis, the rate of included preterm infants, the sample size and the timing of the dosage. Therefore, further studies are needed to confirm and ascertain the additional value of this biomarker, especially to establish reference ranges for those newborns with surgical diseases, renal failure, congenital heart disease, perinatal asphyxia and pathological conditions other than sepsis and to rule out the possibility that these conditions influence the threshold of P-SEP.

## Figures and Tables

**Table 1:** Distribution of percentiles of presepsin levels (pg/mL) in term and preterm neonates studied by Pugni et al. (2015) [14].

	*5th Centile*	*25th Centile*	*50th Centile*	*75th Centile*	*95th Centile*
**Term (>37 weeks GA)** **[*n*= 484]**	315	466	604	791	**1178**
**Preterm (24–36 weeks GA)** **[*n*= 195]**	352	503	620	864	**1370**

**Table 2:** Qualitative synthesis of available studies on reference ranges of P-SEP levels in neonatal age and on P-SEP levels in case of early-onset sepsis (EOS), late-onset sepsis (LOS) and both (EOS and LOS together).

Type of Study	Authors, Year	Country	Population	Cut-off Value of P-SEP (pg/mL)	AUC	Type of Sample	Assay	Conclusions
**Reference** **ranges**								
Prospective	Pugni et al., 2015 [14]	Italy	484 healthy term 195 healthy preterm (GA 24–36 w)	604 (*50th centile*) 620 (*50th centile*)	N/A	Whole blood	CLEIA	P-SEP levels are higher in preterm than in at-term neonates
Prospective	Ishii et al., 2018 [15]	Japan	30 healthy term (GA > 37 w)	318.5 *(at birth)* 180.5 *(day 5)*	N/A	Whole blood	CLEIA	P-SEP levels show physiological variation during early neonatal period
Prospective	Poggi et al., 2020 [4]	Italy	183 preterm (GA < 32 w)	583 (0–6 h) 614 (12 h) 604 (24 h) 513 (48 h) *50th centile*	N/A	Whole blood	CLEIA	P-SEP levels in preterm <32 weeks GA are affected by GA during first 24 h of life
**EOS**								
Case- control	Motalib et al., 2015 [16]	Egypt	28 EOS 34 healthy	672	0.95	Serum	CLEIA	P-SEP is an accurate marker in detecting EOS
Prospective	Ozdemir et al., 2016 [17]	Turkey	29 EOS 40 healthy (term neonates)	539	0.77	Serum	CLEIA	P-SEP may be used a reliable and accurate marker for both diagnosis and follow-up of EOS
Case- control	Montaldo et al., 2017 [10]	Italy	32 EOS 38 healthy (GA < 34 w)	788	0.97	Serum	CLEIA	P-SEP is significantly higher in preterm infants with EOS compared with uninfected infants
Prospective	Seliem and Sultan, 2018 [18]	Egypt	76 EOS 212 healthy (GA 24–36 w)	2231	N/A	Umbilical cord blood	ELISA	Umbilical cord blood P-SEP is a predictor of EOS in preterm infants born to mothers with premature rupture of membranes
**LOS**								
Prospective	Poggi et al., 2015 [1]	Italy	19 LOS 21 healthy (GA < 32 w)	885	0.97	Whole blood	CLEIA	P-SEP is an accurate marker for the diagnosis of LOS in preterm infants
Case- control	Sabry et al., 2016 [19]	Egypt	80 LOS 40 healthy	722	0.99	Serum	ELISA	P-SEP is an accurate marker for the diagnosis of LOS
Prospective	Topcuoglu et al., 2016 [20]	Turkey	42 LOS (GA < 34 w)	800.5	0.86	Plasma	CLEIA	P-SEP can used as a reliable biomarker for the diagnosis of and response to treatment in LOS
Prospective	Astrawinata et al., 2017 [21]	Indonesia	40 LOS in preterm 40 healthy	406	0.89	Whole blood	CLEIA	P-SEP is the earliest and best-performing marker of LOS for the prognosis of preterm neonatal mortality when compared to CRP and PCT
**EOS/LOS**								
Prospective	Mussap et al., 2015 [22]	Italy	25 sepsis 25 SIRS 25 healthy	600	0.99	Whole blood	CLEIA	In critically ill neonates, P-SEP could help in diagnosis and follow-up of neonatal sepsis and non-bacterial SIRS
Case- control	Saied Osman et al., 2015 [23]	Egypt	40 sepsis 15 healthy (full-term)	875	0.95	Plasma	CLEIA	P-SEP is a novel diagnostic marker in neonatal sepsis
Prospective	Mostafa et al., 2015 [24]	Egypt	49 sepsis 29 healthy	686	0.78	Plasma	CLEIA	P-SEP is useful in neonatal sepsis
N/A	Tabl et al., 2016 [25]	Egypt	22 sepsis 28 non- infectious SIRS 20 healthy (term neonates)	812	0.99	Plasma	CLEIA	P-SEP can discriminate between infections and non-infectious inflammatory conditions
Prospective	Xiao et al., 2016 [26]	China	140 sepsis 53 healthy	786	0.94	Whole blood	CLEIA	PSEP is accurate in early identify neonatal hematosepsis; its diagnostic value is superior to other laboratory Biomarkers
Prospective	Miyosawa et al., 2018 [27]	Japan	13 sepsis 18 healthy (preterm)	795	0.86	Whole blood	CLEIA	P-SEP can discriminate between infections and non-infectious inflammatory conditions
Prospective	Kumar, et al., 2018 [28]	India	41 sepsis	1800	0.90	Plasma	CLEIA	P-SEP, in comparison with CRP and PCT, offers a better sensitivity and negative predictive value
Prospective	Iskandar et al., 2018 [29]	Indonesia	51 sepsis	706.5	0.80	Whole blood	CLEIA	In early diagnosis of neonatal sepsis, compared with procalcitonin, presepsin seems to provide a better diagnostic value
Prospective	Hashem et al., 2020 [30]	Egypt	133 sepsis 102 healthy	686	0.88	Plasma	CLEIA	Presepsin is a valuable early diagnostic and monitoring sepsis biomarker, with higher specificity compared to neutrophil CD64 (nCD64)
Prospective	Pietrasanta et al., 2021 [31]	Italy	58 infections 77 sepsis 24 septic shock	987.5	0.86	Whole blood	CLEIA	P-SEP is an early marker of neonatal sepsis severity, but does not support the early identification of neonates with positive blood culture

N/A: Not available. CLEIA: chemiluminescence immune-assay. ELISA: enzyme-linked immunosorbent assay.

**Table 3:** Qualitative synthesis of four meta-analyses available in previous research on P-SEP in neonatal age.

Number of Studies Considered	Authors, Year	Country	Number of Infants Included	Cut-off Value of P-SEP (pg/mL)	AUC	Pooled Sensitivity	Pooled Specificity	Conclusions
**11 studies**	Bellos et al., 2018 [32]	Greece	783 (391 sepsis vs. 392 controls)	<650	0.96	91%	85%	Diagnostic accuracy of P-SEP resulted high in detecting neonatal sepsis
				650–850	0.99	91%	97%	
				>850	0.97	90%	86%	
**28 studies comparing CRP, PCT, CRP + PCT or P-SEP**	Ruan et al., 2018 [33]	China	2661 (1281 sepsis vs. 1380 controls)	722	0.99	94%	98%	The combination of PCT and CRP or presepsin alone improves the accuracy of diagnosis of neonatal sepsis
**10 studies**	Van Maldeghem et al., 2019 [34]	Holland	1369 (89 EOS, 61 LOS, 209 EOS and LOS combined vs. 1010 controls)	305–672	0.94	81%	86%	P-SEP is a promising and rapid-responding diagnostic biomarker for EOS and LOS. The difference in pooled means between EOS and LOS underlines the importance of considering them as two different disease entities
				801–855	N/A	81%	100%	
**9 studies**	Parri et al., 2019 [35]	Italy	3 studies including 268 infants	<600	0.81	93%	81%	Even though it cannot be recommended as a single diagnostic test, P-SEP could be a helpful and valuable biomarker in neonates with suspected sepsis
			6 studies including 375 infants	>600	0.97	87%	100%	

## Data Availability

Not applicable.
